# Impact of utilisation of uncompleted handouts on power point presentations (PPT) in rural Indian medical institute

**Published:** 2016-07

**Authors:** ROSHAN BHAISARE, BHAVNA KAMBLE

**Affiliations:** 1 Department of Orthopaedics, Jawaharlal Nehru Medical College, Sawangi (M) Wardha, Maharashtra, India;; 2 Department of Oto-Rhinolaryngology, Jawaharlal Nehru Medical College, Sawangi (M) Wardha, Maharashtra, India

**Keywords:** Education, Medical education, Undergraduate, Lecture, Lecture notes

## Abstract

**Introduction:**

Note taking while attending a PPT requires high activity of memory and writing process which ultimately leads to what is called “death by power point” referring to boredom and fatigue.  To overcome this we planned to evaluate the impact of utilisation of uncompleted handouts given prior to PPT presentations.

**Methods:**

Final year MBBS students were divided in 2 batches, batch A and batch B.  For a set of lectures one batch was provided with handouts before lecture while the other batch was given lectures only. Crossover was done to avoid bias, all the lectures being given by the same presenter.  At the end of each lecture, a short questionnaire of 10 Multiple Choice Question (MCQ) was provided to the students. Mean scores were calculated for lectures with handouts and without handouts.

**Results:**

For a set of lectures, when batch A was provided with handouts, the mean score was 28.2; for batch B to which no handouts were given the mean score was 23.4. Similarly, for batch B when provided with handouts the mean score was 29.1, for batch A which was not provided with handouts the mean score was 24. There was an average increase of 4.2 marks. Actual gain when handouts were provided was 1.2 marks per lecture.  It was more for the batch comprising of repeater students as compared to the batch of fresher students. Increase in attendance was also noted.

**Conclusion:**

Providing uncompleted handouts before a didactic lecture definitely results in increase in knowledge gain; repeater students benefit more with uncompleted handouts,

## Introduction

Power point presentation is the preferred method of visual aid in lecture halls (1).It is the main instructional medium used at many universities (2). Power point presentations have definite advantages over the traditional chalk board, and overhead projectors (3, 4). But note taking in power point based class is difficult due to the speed of presentation (2). Many times in power point presentation more emphasis is given over format than content (5).Note taking while attaining a power point presentation requires high activity of memory and writing process, which leads to relatively high mental and psychotic load that results in boredom during presentation (6). This may ultimately lead to what is called “death by power point” referring to the status of boredom and fatigue and attention loss (5). As the attention span of students is decreasing gradually, power point presentations are becoming boring to the students. Hence the resultant output of gain of knowledge is decreasing. To overcome this, many educationalists have proposed different modules. Some universities provide comprehensive notes; but these full notes might have a negative effect on active attendance of the class (2). Providing classroom handouts may help to increase the attention span and knowledge gain and that is why classroom handouts are a very sensitive topic among education professionals (7).

There are different types of classroom handouts; one type is providing a copy of the slides as it is. This type of handout is inflexible. Another type of handout is a structured outline of the lecture. In this type of handout plenty of space for note taking is provided, but the limiting factor is speed of delivery of the lecture.  The third type is uncompleted handouts with diagrams and figures that students will complete with lecture. Advantage of this handout is keeping the students involved in lecture and saving their time since they don’t have to constantly write as the presenter talks. Another important aspect is when to provide handouts. Generally, they are provided at the start or end of a lecture. Handouts at the start of lectures are preferred (8).

In this study we evaluated the impact of utilisation of uncompleted handouts given one day prior to the actual didactic lecture to the final year MBBS students of a deemed university. We evaluated the impact by calculating the score of a short questionnaire of Multiple Choice Questions from the same topic. The questionnaire was given at the end of the lecture.  

## Methods

The study was conducted in a Rural medical college set up. Students of final year bachelor of medicine and bachelor of surgery (MBBS) were the participants of the study. Prior to the study, the institutional ethical committee approval was taken. The study was carried out during the didactic lectures on orthopaedics based on the undergraduate curriculum of the university. The study was done with the aim to analyse the impact of using uncompleted handouts prior to didactic lecture (power point presentation).

The design of study was interventional, using 2 groups and post tests.  It was a prospective study. 180 students of 7^th^ semester were divided into two groups: A and B. This division was actually done according to the college undergraduate lecture roaster.  Group A consisted of 77 students (all fresher) and group B consisted of 103 students (77 fresher + 26 repeaters).

Before starting the study, uncompleted handouts of the didactic lectures were prepared in consultation with peers. The finalised handouts were printed. Similarly, a short questionnaire of 10 questions (MCQs) was prepared on each topic, reviewed by peers.  Hard prints of the questionnaire were prepared. The batch representatives were given the responsibility of distributing handouts to the students a day prior to the actual lecture. In   the university undergraduate curriculum, one orthopaedic lecture was scheduled every week. Two presenters were assigned to each one of the two batches for a single topic at two different lecture halls. In order to avoid bias, all the lectures under study were taken by a single presenter. The lectures were delivered using power point presentations (PPT).  Each week one batch was involved in the study and the other batch was not. Another presenter delivered lectures as per university curriculum to the non-involved group. Hence, the study did not interfere with the schedule of the lectures.   The lecture duration was one hour out of which nearly 52 minutes were allocated to actual lecture and 8 minutes to recording the attendance of the students.  During this attendance time students were asked to complete the short questionnaire; hence, extra time was not needed. 

In order to avoid intervention bias, power point presentations were delivered by the same presenter. Hence, one batch was given a lecture on one topic this week and next week the other batch was given the lecture on the same topic by the same presenter. To avoid sampling bias, for the first week intervention batch was selected randomly by draw of lots. There was a possibility that students may exchange handouts to the other batch which may adversely affect the study. Therefore, hand outs were provided to the 2^nd^ batch on the 2^nd^ and 4^th^ weeks.  In the first week, batch B was selected by draw of lots and they were not provided with handouts. After the PPT, at the end of the session the questionnaires were distributed and after completion by the students, they were collected.  In next week, batch A was provided with handouts a day prior to PPT   and at the end of the session the same questionnaires were distributed and collected .This batch (batch A)   was not provided with handout on the 3^rd^ week. In the fourth week, the other batch (batch B) was provided with handout a day prior to the lecture. By this we have achieved crossover as well as confidentiality of our handouts. The same presenter delivered the same lecture (PPT) on the same topic to both batches. The investigator checked the questionnaires, and calculated the average score for each lecture.

This cycle was repeated for all 16 lectures covering 8 topics over four months.  That means out of 8 lectures for each batch, four were with handouts and four were without handouts. 

## Results

For the mean score of each batch, with or without handouts see (Table 1). Intra-batch results (Table 2) shows the gain of each group when they were provided with handouts.

The total average score for (PPT ONLY) was 23.7. The total average score for (UNCOMPLETED HANDOUTS + PPT) was 28.7. By unpaired t-test, for the group with handouts the mean was 7.163±0.453 and for group with no handout the mean was 5.925±0.417. The two-tailed P-value was less than 0.0001 and this difference is considered to be extremely statistically significant. 

**Table 1:** Average score, each batch, with or without handouts

**Lecture**	** Handouts **			
	Yes		No	
	A	B	A	B
**Lecture 1**	7.2			5.9
**Lecture 2 **		6.9	5.5	
**Lecture 3**	7.6			5.8
**Lecture 4**		6.8	5.7	
**Lecture 5**	6.6			5.4
**Lecture 6**		7.6	5.9	
**Lecture 7**	6.8			6.3
**Lecture 8**		7.8	6.9	
**Total score**	28.2	29.1	24	23.4

**Table 2:** Scores (Intra-batch) and gain

**Handouts **	**Batch A**	**Batch B**	**Average **
**No**	24	23.4	23.7
**Yes **	28.2	29.1	28.7
**Gain **	4.2	5.7	4.8
**Average **	1.05	1.42	1.2

The average gain of both batches for 4 cycles comprising of 8 lectures when handouts were provided was 4.8 (Table 2).  Hence, the net gain was 1.2 marksper lecture.  For batch A, when provided with handouts, the gain per lecture was 1.05 marks. For batch B, when provided with handouts, the gain per lecture was 1.4 marks. The gain was more for batch B as compared to batch A. The probable explanation for this could be that batch B comprised of fresher and repeater students as compared to batch A which included fresher students only.  Providing handouts may actually have helped the repeater students to gain more knowledge.

## Discussion

During a didactic lecture by power point presentation, the instructor should actively engage the students in the process of conveying information. One strategy is providing handouts. In this approach, students are involved in note taking which requires more focus. This strategy ultimately leads to improved learning outcomes (9-11). George Browni & Michael Manogue stressed out the fact that, if a lecture is used to cover fact, finding  a good textbook can do it much better. They have pointed out that lectures do have limitations and at times lectures can be boring, worse and useless. They have suggested that innovative methods of lecture presentation and providing handouts definitely helps (12).

There are various types of handouts. One type is the direct print of the power point slides. The advantage of this kind of handout is, that it is easy to prepare. The disadvantage is that the student is getting a complete print of the slides and this may adversely affect his/her class attendance.  Another type of handout is a structured outline of the lecture in which a plenty of space is kept for note taking. Advantage of this type of handout is that, it is structured and the disadvantage is that students still have to write a lot.  The third type of handout is uncompleted handouts; this type of handout has notes, diagrams and figures and student should complete them as the lecture proceeds. The most important advantage of this handout is that the students are more involved during the class since they do not have to write continuously (8).

With uncompleted handouts, students need to write only minimal notes and hence they can focus more on the content of PPT. Also they have more time to listen and focus on the educational content. If the concept is ambiguous, the student can record brief explanation. Taking notes by each student differs from the others since each student’s requirements are different. Moreover, students’ personal notes help them for subsequent recall. A study by Larsen in 2009 indicated that proper use with a perfect design of the handout increases short time recall (13). Handouts are written material which are helpful to the teacher as well as the students. Handouts of slides allow students to participate more in thinking about the concepts rather than writing down every word of the lecture. The literature on handouts shows that students acquire higher scores in tests (14).

In a study by N. Hephzibah Kirubamani, he concluded that since handouts are portable it will improve recall of information.  Students give positive feedback for effectiveness of the handouts.  He also stressed that much care in needed in preparing the handout. A handout which is rich in content and information level makes the students learn from handout later on their own and better than students’ notes. In his study 94% of the students expressed that they could use handouts as a guide for future learning (15). Brown & Atkins suggests that it is the interest but not the aim to produce marked changes in achievement which is more likely to influence the students’ attitude towards the subject. Hence, making a lecture interesting is very important and providing a handout could actually help (16).

Moreover these handouts could give the students a general overview of the educational material. With handouts in hand, students at any time can return to the previous slides thus preventing missing the past concepts during presentation. Jannis Mitkits in a research paper “The Use of Classroom Handouts” concluded that handouts are very useful for the learning process (7). At the same time the author had put the responsibility on the teachers to evaluate the cost and benefits to the students’ development. Handouts are a valuable aid and guide to the students to study in an organized way. It also helps them to cope with their stress during exams (17).

In the present study when for a set of lectures batch A was provided with handouts prior to the lecture, the average score was 28.2 as compared to batch B which was not provided with handouts, for whom the score was 23.4.  After crossover for the other set of lectures where batch B was provided with handouts the average score was 29.1 as compared to batch A which was 24 without handouts. The total average score for PPT ONLY was 23.7 and for (UNCOMPLETED HANDOUTS + PPT) was 28.7. There was a net gain of 4.8 marks, for 4 episodes comprising of 8 lectures. 

When the scores were compared between the fresher and repeater students, the gain was more for batch B (5.7) as compared to batch A (4.2). The probable explanation for this could be that batch B was comprised of fresher and repeater students as compared to batch A which included fresher students only.  Providing handouts may actually have helped the repeater students to gain more knowledge. 

The improvement in attendance was also observed during the study. Since this is short term study we cannot authoritatively say that this improvement was due to the innovation in the teaching method. 

The present study proposes that providing uncompleted handouts before didactic lectures definitely results in increase in knowledge gain. Also this is more helpful for the repeater students; it also helps in increasing the attendance of the class.

This strategy (using uncompleted handouts) could be very useful in developing countries where there are limited medical teachers and the teachers need to handle many tasks at the same time apart from teaching. This could be of extreme help to the medical teachers who are physicians, too.

**Limitations**

This study was of short duration and was limited to only one medical college and that to a single subject. The present study was also of short outcome in indicator matrix, so for further intermediate outcomes, we would like to expand the study to other subjects and other colleges of the university for longer duration. 

## Conclusion

From this study it can be concluded that providing uncompleted handouts before didactic lectures definitely results in increase in knowledge gain. Repeater students benefit more with uncompleted handouts. The association between improvement in attendance and uncompleted handouts could not be established conclusively. 
